# The effect of selective cerebral perfusion on cerebral versus somatic tissue oxygenation during aortic coarctation repair in neonates and infants

**DOI:** 10.1186/s12871-021-01498-0

**Published:** 2021-11-15

**Authors:** Li Zhang, Lu Liu, Zhiqiu Zhong, Hengfang Jin, Jian Jia, Lingzhong Meng, Xuming Mo, Xiaohua Shi

**Affiliations:** 1 Department of Anesthesiology, Children’s Hospital of Nanjing Medical University, 210008, Nanjing, Jiangsu Province China; 2 Department of Anesthesiology, Yale University School of Medicine, 06520 New Haven, CT USA; 3 Department of Cardiothoracic, Children’s Hospital of Nanjing Medical University, 210008 Nanjing, Jiangsu Province China

**Keywords:** Neonates and infants, Aortic coarctation repair, Cerebral tissue oxygen saturation, Somatic tissue oxygen saturation, prospective cohort study

## Abstract

**Background:**

Suboptimal tissue perfusion and oxygenation may be the root cause of certain perioperative complications in neonates and infants having complicated aortic coarctation repair. Practical, effective, and real-time monitoring of organ perfusion and/or tissue oxygenation may provide early warning of end-organ mal-perfusion.

**Methods:**

Neonates/infants who were scheduled for aortic coarctation repair with cardiopulmonary bypass (CPB) and selective cerebral perfusion (SCP) from January 2015 to February 2017 in Children’s Hospital of Nanjing Medical University participated in this prospective observational study. Cerebral and somatic tissue oxygen saturation (SctO_2_ and SstO_2_) were monitored on the forehead and at the thoracolumbar paraspinal region, respectively. SctO_2_ and SstO_2_ were recorded at different time points (baseline, skin incision, CPB start, SCP start, SCP end, aortic opening, CPB end, and surgery end). SctO_2_ and SstO_2_ were correlated with mean arterial pressure (MAP) and partial pressure of arterial blood carbon dioxide (PaCO_2_).

**Results:**

Data of 21 patients were analyzed (age=75±67 days, body weight=4.4±1.0 kg). SstO_2_ was significantly lower than SctO_2_ before aortic opening and significantly higher than SctO_2_ after aortic opening. SstO_2_ correlated with leg MAP when the measurements during SCP were (*r*=0.67, *p*<0.0001) and were not included (*r*=0.46, *p*<0.0001); in contrast, SctO_2_ correlated with arm MAP only when the measurements during SCP were excluded (*r*=0.14, *p*=0.08 vs. *r*=0.66, *p*<0.0001). SCP also confounded SctO_2_/SstO_2_’s correlation with PaCO_2_; when the measurements during SCP were excluded, SctO_2_ positively correlated with PaCO_2_ (*r*=0.65, *p*<0.0001), while SstO_2_ negatively correlated with PaCO_2_ (*r*=-0.53, *p*<0.0001).

**Conclusions:**

SctO_2_ and SstO_2_ have distinct patterns of changes before and after aortic opening during neonate/infant aortic coarctation repair. SctO_2_/SstO_2_’s correlations with MAP and PaCO_2_ are confounded by SCP. The outcome impact of combined SctO_2_/SstO_2_ monitoring remains to be studied.

## Introduction

Coarctation of the aorta (CoA) is a congenital narrowing of upper descending thoracic aorta adjacent to the site of attachment of ductus arteriosus just distal to the left subclavian artery. The reported prevalence of CoA is approximately 4 per 10,000 live births [1, 2] or 400 per million live births, [3] which accounts for ~4 % of all congenital heart defects [3]. The preferred treatment for native CoA during childhood is surgical correction based on the considerations related to midterm and long-term outcomes in many children’s hospitals [4, 5]. The optimal age for elective repair of aortic coarctation is controversial. Some centers perform elective aortic coarctation repair around 1.5 years of age [6]. However, surgery at a much earlier age may be required depending on the severity of the coarctation and the adequacy of the collateral flow.

CoA does not cause hemodynamic problems in utero, as two-thirds of the combined cardiac output flows through the patent ductus arteriosus (PDA) into the descending thoracic aorta, bypassing the site of constriction at the isthmus. During the neonatal period, when the PDA and foramen ovale begin to close, the cardiac output that must cross the narrowed aortic segment to reach the lower extremities steadily increases and the clinical consequences start to show. The central dilemma of this unique congenital malformation is the divergent hemodynamic patterns in the body parts that are perfused by the vasculature proximal and distal to the coarctation, respectively, i.e., systolic hypertension in the upper extremities while hypotension in the lower extremities.

CoA repair in small children has inherent risks [7]. The operative death in children less than one-year-old can be as high as ~40 %, albeit that in children older than one year is much less (~0.4 %).^7^ Suboptimal organ perfusion and tissue oxygenation as a result of cardiovascular malformation, cardiopulmonary bypass (CPB), deep hypothermic circulatory arrest, and selective cerebral perfusion (SCP) may be among the root causes of end-organ injuries in this patient population. The ability to monitor real-time organ/tissue perfusion and oxygenation can facilitate intraoperative management and reduce complications.

A tissue oximeter based on near-infrared spectroscopy (NIRS) can non-invasively and continuously measure the hemoglobin saturation of the mixed arterial, capillary, and venous blood in the tissue bed that is about 1-2.5 cm underneath the sensor [8]. Cerebral tissue oxygen saturation (SctO_2_) is measured with the sensor placed on the forehead, while somatic tissue oxygen saturation (SstO_2_) is measured with the sensor positioned at a peripheral location. NIRS-measured tissue oxygenation is determined by the balance between tissue oxygen consumption and supply; when tissue oxygen consumption remains relatively stable, it is essentially determined by tissue perfusion or oxygen delivery [9].

We hypothesize that tissue beds perfused by the vasculature proximal and distal to the aortic coarctation have distinct oxygenation patterns during aortic coarctation surgery in neonates and infants. This study aims to compare SctO_2_ and SstO_2_ monitored on the forehead and at the paraspinal thoracolumbar region, respectively, and correlate SctO_2_/SstO_2_ with relevant physiological variables.

## Methods

This prospective cohort study was approved by the Internal Review Board at Children’s Hospital of Nanjing Medical University located in Nanjing, Jiangsu Province, China. Consent for study participation was obtained from the patient’s guardians before surgery.

### Patients

Neonates and infants undergoing complicated aortic coarctation repair surgery from January 2015 to February 2017 in the Children’s Hospital of Nanjing Medical University participated in this study. The inclusion criteria were age < 1-year-old and coarctation surgery mandating CPB and SCP. The exclusion criteria were emergent surgery and refusal by the patient’s guardians.

### Anesthesia

After arriving in the operating room, patients were first sedated using intravenous fentanyl (2 mcg/kg) and midazolam (0.05 mg/kg). Standard electrocardiography, pulse oximetry, and non-invasive blood pressure monitoring, in addition to SctO_2_/SstO_2_ monitoring, were applied before anesthesia induction. Anesthesia was induced using intravenous administration of penehyclidine (0.01 mg/kg), fentanyl (10 mcg/kg), midazolam (0.1 mg/kg), and rocuronium bromide (0.6 mg/kg). All patients were intubated and mechanically ventilated with a tidal volume of 6-8 ml/kg, inspiratory to expiratory time ratio of 1:1.5-2, and inspired oxygen of 40 %. The respiratory rate was adjusted per the level of end-tidal carbon dioxide which was maintained at 45-50 mmHg before CPB in patients with preductal patent ductus arteriosus. An arterial catheter (22-24 GA) was placed in the right radial and femoral arteries to monitor the arterial blood pressure on the right arm and right leg, respectively. A central venous catheter (5 F) was placed through the right jugular vein for intravenous access and central venous pressure monitoring. Anesthesia was maintained using sevoflurane inhalation and intravenous infusion of rocuronium bromide (0.1 mg/kg/h), in addition to the intermittent intravenous bolus of fentanyl (10~20 mcg/kg), and midazolam (0.1 mg/kg).

### Tissue Oxygenation Monitoring

SctO_2_ and SstO_2_ were monitored by a tissue oximeter (CASMED MC-2030 C, CAS Medical System, Branford, CT). SctO_2_ was monitored with the pediatric sensor placed on the right forehead, while SstO_2_ was monitored with the pediatric sensor placed on the right paraspinal muscle at the T_10_-L_2_ level. The first set of SctO_2_ and SstO_2_ measurements were obtained with the patient sedated but spontaneously breathing (i.e., before anesthesia induction). SctO_2_ and SstO_2_ were then measured at the pre-determined time points throughout the surgery.

### Surgery

A midline sternal incision was made for access. The ascending aorta and superior and inferior vena cava were cannulated in 20 patients. One patient with an interrupted aortic arch received additional pulmonary artery cannulation. Following heparinization, CPB was established using an artificial heart-lung machine (Maquet HL 20, MAQUET Cardiovascular, LLC, Wayne, NJ, USA) with a membrane oxygenator (D901, Sorin Group Italia S.r.I., Mirandola, Italy). The CPB started with moderate to low temperature (24-28 °C) and moderate to high flow rate (100-150 ml/kg/min). Following the clamping of the aorta and superior and inferior vena cava, the heart was arrested using histidine-tryptophan-ketoglutarate solution (Dr. Franz Köhler Chemie GmbH, Bensheim, Germany) infused at the aortic root. Following the correction of intracardiac malformation, the rectal temperature was further decreased to 18-20 °C for aortic arch repair. SCP was accomplished following the advancement of the aortic cannula into the innominate artery with a flow rate of 20-40 ml/kg/min. The successful coarctation repair was followed by aorta re-opening and then the termination of CPB. Patients were mildly hyperventilated, and the hemodynamics were normally supported using epinephrine 0.05-0.1 mcg/kg/min and milrinone 0.5 mcg/kg/min.

### Data Collection

Physiological measurements including mean arterial pressure (MAP), partial pressure of arterial blood carbon dioxide (PaCO_2_), hematocrit, temperature, arterial blood oxygen saturation (SaO_2_), mixed venous blood oxygen saturation (SmvO_2_), SctO_2_ and SstO_2_ were prospectively recorded at the pre-determined time points. These time points were baseline with the patient sedated and spontaneously breathing, skin incision, CPB start, SCP start, SCP end, aortic opening, CPB end, and surgery end. 1 ml of arterial blood and 1 ml of mixed venous blood were taken for blood gas analysis at each time point.

### Statistical Analysis

Data were presented in mean ± SD. The difference between two variables at different time points was assessed using paired t-test. Pearson’s correlation coefficient was used to assess the correlation between different variables. A two-sided p-value < 0.05 was regarded as statistically significant.

## Results

Among the 21 patients who participated in this study, 16 were males, 3 neonates (< 30 days old), and 18 infants (< 1-year-old). The average age was 75 ± 67 days (range = 12 to 333 days). The average body weight was 4.4 ± 1.0 kg (range = 2.8 to 6.5 kg) (Table 1). All patients had ventricular septal defect; 20 patients had patent ductus arteriosus; 16 had pulmonary hypertension; and 9 had an atrial septal defect.

**Table 1:** Patient demographics

Patient #	Gender	Age (Day)	Weight (kg)	PDA	VSD	ASD	Pulmonary hypertension	Others	ASA Grade
1	Female	79	3.7	Y	Y	Y	Y	Aortic arch hypogenesis	III
2	Male	26	2.8	Y	Y	Y	N	Aortic arch hypogenesis	III
3	Male	50	3.8	Y	Y	Y	Y	Aortic arch hypogenesis	IV
4	Male	141	6	Y	Y	N	Y		III
5	Female	38	2.8	Y	Y	Y	Y	Aortic arch hypogenesis	IV
6	Male	22	3.8	Y	Y	Y	Y	Respiratory failure	IV
7	Female	74	5	Y	Y	N	Y		III
8	Male	71	4.5	Y	Y	N	Y		III
9	Male	41	4.5	Y	Y	N	Y	Patent foramen ovale Mitral and tricuspid insufficiency	IV
10	Female	98	4.7	Y	Y	Y	Y		IV
11	Female	333	6.5	Y	Y	N	Y		IV
12	Male	46	3.5	Y	Y	Y	N		IV
13	Male	47	2.8	Y	Y	N	Y	Patent foramen ovale	III
14	Male	83	4.9	Y	Y	N	Y		III
15	Male	53	4	Y	Y	N	Y		III
16	Male	120	5.6	Y	Y	N	N		III
17	Male	92	5.9	Y	Y	N	N	Patent foramen ovale	III
18	Male	52	4.5	Y	Y	N	Y	Taussig-Bing anomaly	IV
19	Male	12	4	Y	Y	Y	N		III
20	Male	49	4.9	Y	Y	N	Y	Patent foramen ovale, anomalous coronary origin	IV
21	Male	48	4.5	N	Y	Y	Y	Interrupted Aortic Arch Rh hemolytic disease	IV

PDA = patent ductus arteriosus; VSD = ventricular septal defect; ASD = atrial septal defect; ASA = American Society of Anesthesiologists; Y = yes; N = no

The CPB, aortic clamping, and SCP time were 185 ± 101 min, 68 ± 17 min, and 23 ± 6 min, respectively. The length of hospital stay, ICU stay, and postoperative intubation time was 25 ± 8 days, 11 ± 2 days, and 6 ± 1 days, respectively.

**Fig. 1:**
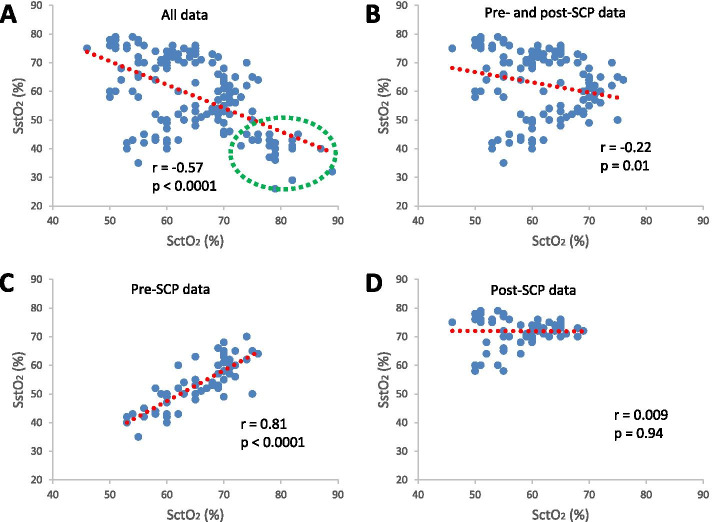
Correlations between cerebral tissue oxygen saturation (SctO_2_) and somatic tissue oxygen saturation (SstO_2_). The correlations were based on all data (**A**), data with the measurements during selective cerebral perfusion (SCP) excluded (**B**), only data before SCP (**C**), and only data after SCP (**D**), respectively. The red dotted lines are trend lines and the data points in green dotted circle are measurements during SCP

The physiological measurements at different time points are presented in Table 2. SstO_2_ was consistently and significantly lower than SctO_2_ at each time points before the aortic opening, including the baseline (∆ ≈ -12 %), skin incision (∆ ≈ -12 %), CPB start (∆ ≈ -12 %), SCP start (∆ ≈ -40 %), and SCP end (∆ ≈ -21 %) (∆ = SstO_2_ – SctO_2_). Following the aortic opening, this relationship was reversed with the SstO_2_ consistently and significantly higher than SctO_2_ at each time point, including aortic opening (∆ ≈ +24 %), CPB end (∆ ≈ +12 %), and surgery end (∆ ≈ +8 %). Overall, SctO_2_ and SstO_2_ were significantly correlated (*r* = -0.57, *p*<0.0001, Fig. 1 A); however, the correlation became weaker after exclusion of the measurements during SCP (*r* = -0.22, *p* = 0.01, Fig. 1B). The correlation between SctO_2_ and SstO_2_ was at its strongest when only considering the data before SCP (*r* = 0.81, *p* < 0.0001, Fig. 1 C) and weakest when only considering the data after SCP (*r* = 0.009, *p* = 0.94, Fig. 1D).

**Table 2:** Physiological variables measured at different time points

Variable (unit)	Baseline^a^	Skin incision	CPB start	SCP start	SCP stop	Aorta open	CPB stop	Surgery end
SctO_2_ (%)	70.3 ± 3.5	67.6 ± 3.5	58.6 ± 3.5	79.6 ± 4.0	71.8 ± 2.5	51.8 ± 2.3	59.0 ± 2.8	61.3 ± 6.0
SstO_2_ (%)	58.8 ± 5.8	55.1 ± 6.2	46.4 ± 6.7	39.6 ± 5.2	50.5 ± 4.9	75.8 ± 4.4	70.5 ± 2.3	69.5 ± 6.2
SaO_2_ (%)	99.6 ± 0.4	99.6 ± 0.4	99.6 ± 0.4	99.8 ± 0.2	99.9 ± 0.1	99.6 ± 0.4	99.5 ± 0.4	99.4 ± 0.6
SmvO_2_ (%)	70.7 ± 5.7	68.4 ± 5.2	56.4 ± 5.0	78.3 ± 4.0	74.4 ± 3.4	58.5 ± 7.8	60.3 ± 5.7	62.3 ± 9.1
MAP-arm (mmHg)	65.9 ± 3.8	63.4 ± 3.0	56.4 ± 3.4	57.6 ± 4.5	54.1 ± 2.5	57.3 ± 4.1	60.2 ± 3.9	59.9 ± 5.1
MAP-leg (mmHg)	44.2 ± 8.5	42.4 ± 7.7	38.7 ± 6.7	22.1 ± 4.8	28.1 ± 9.5	45.2 ± 9.5	48.5 ± 9.6	50.7 ± 10.7
PaCO_2_ (mmHg)	45.3 ± 3.8	42.4 ± 3.4	38.9 ± 2.6	31.2 ± 1.8	29.7 ± 1.2	31.6 ± 1.5	35.1 ± 3.5	37.9 ± 3.1
Hematocrit (%)	38.3 ± 2.1	38.0 ± 2.2	37.2 ± 2.1	21.0 ± 1.8	20.6 ± 2.7	25.1 ± 1.8	28.7 ± 1.6	34.3 ± 1.5
Temperature (°C)	37.0 ± 0.5	36.6 ± 0.4	36.2 ± 0.4	20.1 ± 1.2	19.6 ± 1.2	28.6 ± 2.6	36.5 ± 0.5	36.1 ± 0.5
Lactate (mmol/l)	1.3 ± 0.2	1.3 ± 0.3	1.6 ± 0.3	2.5 ± 0.3	3.7 ± 0.4	3.1 ± 0.3	2.6 ± 0.3	2.5 ± 0.4

^a^with patient sedated using small dose of fentanyl and midazolam and spontaneously breathing room air

CPB = cardiopulmonary bypass; SCP = selective cerebral perfusion; SctO_2_ = cerebral tissue oxygen saturation; SstO_2_ = somatic tissue oxygen saturation; SaO_2_ = arterial blood oxygen saturation; SmvO_2_ = mixed venous blood oxygen saturation; MAP-arm = mean arterial pressure measured on arm; MAP-leg = mean arterial pressure measured on leg; PaCO_2_ = arterial blood carbon dioxide partial pressure

**Fig. 2:**
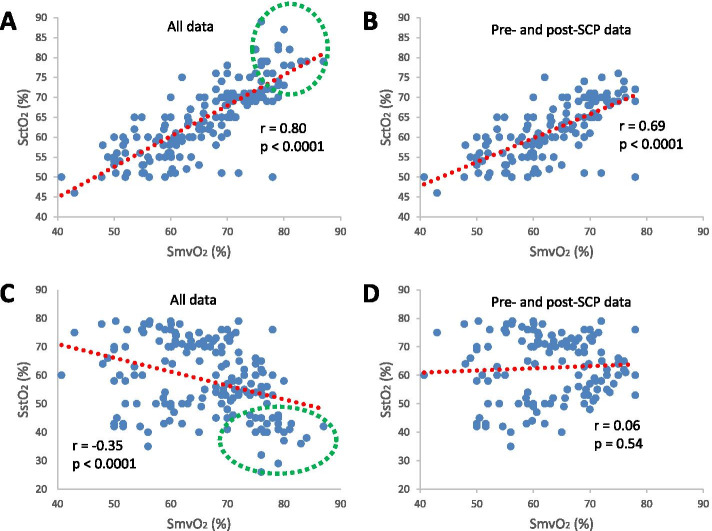
Correlations between mixed venous blood oxygen saturation (SmvO_2_) and cerebral tissue oxygen saturation (SctO_2_) (**A** and **B**) and between SmvO_2_ and somatic tissue oxygen saturation (SstO_2_) (**C** and **D**). The correlations were based on all data (**A** and **C**) and data with the measurements during selective cerebral perfusion (SCP) excluded (**B** and **D**), respectively. The red dotted lines are trend lines and the data points in green dotted circles are measurements during SCP

The correlation between SctO_2_ and SmvO_2_ was stronger than that between SstO_2_ and SmvO_2_. SctO_2_ was significantly correlated with SmvO_2_ with the measurements during SCP considered (*r* = 0.80, *p* < 0.0001, Fig. 2 A) and not considered (*r* = 0.69, *p* < 0.0001, Fig. 2B). SstO_2_ and SmvO_2_ were significantly correlated only when the measurements during SCP included (*r* = -0.35, *p* < 0.0001, Fig. 2 C), not excluded (*r* = 0.06, *p* = 0.54, Fig. 2D).

**Fig. 3:**
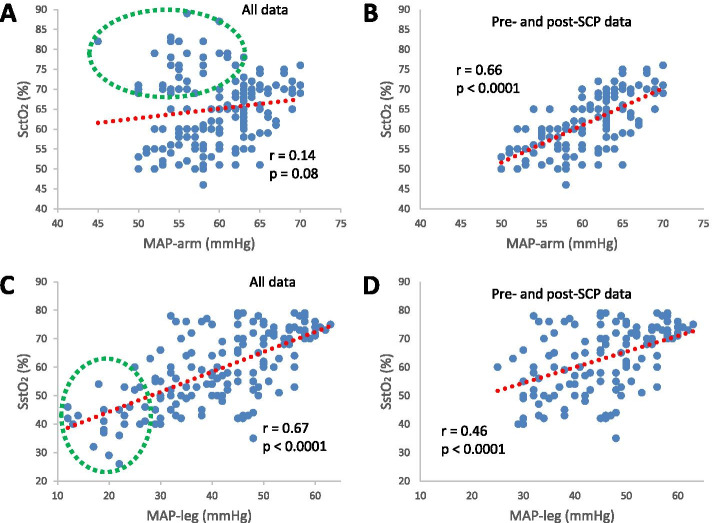
Correlations between mean arterial pressure measured on arm (MAP-arm) and cerebral tissue oxygen saturation (SctO_2_) (**A** and **B**) and between mean arterial pressure measured on leg (MAP-leg) and somatic tissue oxygen saturation (SstO_2_) (**C** and **D**). The correlations were based on all data (**A** and **C**) and data with the measurements during selective cerebral perfusion (SCP) excluded (**B** and **D**), respectively. The red dotted lines are trend lines and the data points in green dotted circles are measurements during SCP

SctO_2_ and MAP measured on the arm were not correlated with all measurements considered (*r* = 0.14, *p* = 0.08, Fig. 3 A); however, they became significantly correlated with the measurements during SCP excluded (*r* = 0.66, *p* < 0.0001, Fig. 3B). In contrast, SstO_2_ and MAP measured on the leg were significantly correlated with measurements during SCP included (*r* = 0.67, *p* < 0.0001, Fig. 3 C) and excluded (*r* = 0.46, *p* < 0.0001, Fig. 3D).

**Fig. 4:**
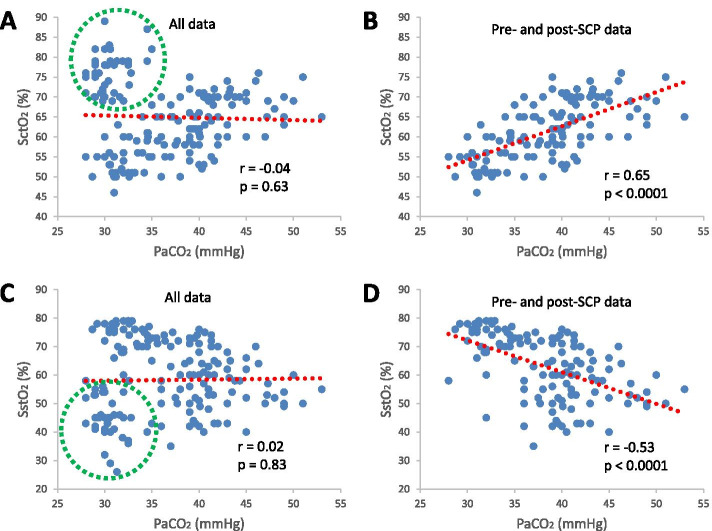
Correlations between arterial blood carbon dioxide partial pressure (PaCO_2_) and cerebral tissue oxygen saturation (SctO_2_) (**A** and **B**) and between PaCO_2_ and somatic tissue oxygen saturation (SstO_2_) (**C** and **D**). The correlations were based on all data (**A** and **C**) and data with the measurements during selective cerebral perfusion (SCP) excluded (**B** and **D**), respectively. The red dotted lines are trend lines and the data points in green dotted circles are measurements during SCP

SctO_2_ and PaCO_2_ were not correlated with all measurements considered (*r*=-0.04, *p* = 0.63, Fig. 4 A); however, they became significantly correlated with the measurements during SCP excluded (*r*=0.65, *p* < 0.0001, Fig. 4B). SstO_2_ and PaCO_2_ were also not correlated with all measurements considered (*r*=0.02, *p* = 0.83, Fig. 4 C); however, they also became significantly correlated with the measurements during SCP excluded (*r* = -0.53, *p* < 0.0001, Fig. 4D). Of note, SctO_2_ positively (Fig. 4B), while SstO_2_ negatively (Fig. 4D), correlated with PaCO_2_ after the exclusion of the measurements during SCP.

## Discussion

Our study showed that the measurements of SctO_2_ and SstO_2_ is surgical stage-dependent in neonates and infants undergoing aortic coarctation repair. SstO_2_ was significantly lower than SctO_2_ before aortic opening; however, following aortic opening, SstO_2_ was significantly higher than SctO2. SctO_2_ and SstO_2_ have distinct correlations with MAP, PaCO_2_, hematocrit, and temperature. SstO_2_ consistently correlates with leg MAP; however, the correlation between SctO_2_ and arm MAP is confounded by the measurements during SCP. The measurements during SCP also confound the correlations between SctO_2_ and PaCO_2_ and between SstO_2_ and PaCO_2_. SctO_2_, not SstO_2_, is significantly correlated with SmvO_2_.

Aortic cross-clamping is a well-known risk factor for cerebral, spinal cord, pulmonary, renal, and visceral injuries [10–12]. The disturbance of systemic and regional blood flow threatens organ perfusion, with the resultant tissue hypoperfusion and overzealous perfusion likely being the root cause of certain perioperative complications. Practical, effective, and real-time monitoring of organ perfusion and/or tissue oxygenation may provide early warning of end-organ mal-perfusion and thereby improve patient outcomes.

Tissue NIRS was originally used to monitor cerebral tissue bed. The recent technological advancement made the monitoring of non-cerebral tissue beds possible [8]. The combined monitoring of both cerebral and non-cerebral tissue beds has the potential to further improve outcomes because the brain and non-brain organs have different schemes of metabolic demands and compensatory mechanisms during hemodynamic instability. Hoffman et al. pioneered the clinical application of multisite tissue oxygenation monitoring in pediatric cardiac patients. They monitored both cerebral oxygenation and renal oxygenation (with the sensor placed over the kidney region) in neonates undergoing stage 1 palliation for hypoplastic left heart syndrome [13]. Berens et al. investigated the clinical application of the same multisite monitoring protocol in 26 pediatric patients (11 neonates, 5 infants, and 10 children) undergoing aortic coarctation repair [14]. They reported that the decline in SstO_2_ during aortic cross-clamping was larger in neonates and infants than children, which was attributed to a better established collateral circulation around the incomplete aortic obstruction in older patients. However, they did not specifically compare SctO_2_ and SstO_2_ at different time points and correlate SctO_2_/SstO_2_ with different physiological parameters.

The significantly lower SstO_2_, compared with SctO_2_, before aortic opening reflects the hypoperfusion of the tissue beds that were perfused by the vasculature distal to the aortic stenosis. The start of CPB led to simultaneous decreases of both SctO_2_ and SstO_2_, likely secondary to decreased global tissue oxygen delivery (delivery = flow*hemoglobin*arterial blood oxygen saturation) assuming tissue oxygen consumption remained unchanged. The start of SCP led to a distinct increase of SctO_2_ while a decrease of SstO_2_, which reflects the effectiveness of SCP in increasing cerebral oxygen delivery and the corresponding worsening of peripheral oxygen delivery. The discrepancy between SctO_2_ and SstO_2_ was lessened toward the end of SCP, which was likely secondary to the adjustment of regional vascular resistance and flow redistribution. The aortic opening led to a remarkable increase of SstO_2_ while a remarkable decrease of SctO_2_, a distinct change in the oxygenation pattern that is likely caused by the hyperperfusion of the distal tissue beds and the simultaneous relative hypoperfusion (or a “steal” phenomenon) of the proximal tissue beds (in relevance to the aortic stenosis). The remarkable SctO_2_ decrease may also relate to the rewarming-related increase in cerebral tissue oxygen consumption. The discrepancy between SstO_2_ and SctO_2_ was gradually minimized thereafter which is likely, again, secondary to the adjustment of the vascular resistance of different tissue beds over time.

The consistent correlation between leg MAP and SstO_2_ reflects the chronic vasodilation and pressure-passive flow in the tissue beds that are perfused by the vasculature distal to the aortic stenosis. The confounding of the correlation between arm MAP and SctO_2_ by the measurements during SCP reflects the overriding effect of SCP on cerebral perfusion [15]. The sloped and linear relationship between arm MAP and SctO_2_ may be a reflection of the loss of cerebral autoregulation in this patient population undergoing the described type of open cardiac surgery [9, 16].

SctO_2_ positively correlated with PaCO_2_, while SstO_2_ negatively correlated with PaCO_2_ when the measurements during SCP are excluded (not included), which again highlights the following: (1) carbon dioxide is a predominantly cerebral vasodilator and hypercapnia leads to increased cerebral perfusion, (2) SCP has an overriding effect on cerebral perfusion, and (3) the correlations between SctO_2_/SstO_2_ and PaCO_2_ are confounded during SCP.

Our study demonstrated a significant correlation between SctO_2_ and SmvO_2_, which is supported by some previous reports [17] and refuted by others [18]. We also showed that SctO_2_ is more consistently correlated with SmvO_2_ than SstO_2_. Tissue oxygen saturation is tissue bed specific and measures the hemoglobin saturation of mixed arterial, capillary, and venous blood, while SmvO_2_ is the hemoglobin saturation of the pooled venous blood coming from a variety of tissue beds. These two parameters are different but related. The stronger correlation of SctO_2_, compared with SstO_2_, with SmvO_2_ reflects the dominance of cerebral metabolism and the dominant contribution of cerebral venous blood to the mixed venous blood in this patient population.

Our study has limitations that highlight the need for future studies. One of the important goals of a clinical monitor is to improve patient outcome via monitor-guided care. This is normally done based on randomized controlled trials to understand if the proposed monitor-guided intervention is better than the conventional approach. In order to do so, an interventional protocol needs to be first established. An intervention protocol needs to specify the tissue dys-oxygenation thresholds beyond which corrective measures need to be instituted. It also needs to establish the intervention algorithm with details of when and how to intervene and the priorities, especially considering that tissue oxygenation is determined by multiple yet integrated variables. However, our study neither specifically studied the intervention guided by SctO_2_/SstO_2_ monitoring nor correlated SctO_2_/SstO_2_ measurements with patient outcomes. In addition, future studies need to address the tissue beds that should be prioritized for monitoring and the details of intervention based on multisite monitoring. The sample size of our study is small. Large-scale studies are needed to validate our findings, establish the intervention protocol, and prove the outcome benefits.

## Conclusions

In summary, SctO_2_ and SstO_2_ have distinct patterns of change during neonate and infant aortic coarctation repair. SctO_2_ and SstO_2_ measurements are surgical stage dependent. The correlations of tissue oxygenation with MAP and PaCO_2_ are confounded during SCP. The distinct changes in SctO_2_ and SstO_2_ and their correlations with MAP and PaCO_2_ suggests the potential value of multisite tissue oxygenation monitoring during high-risk pediatric cardiac surgeries.

## Data Availability

All data generated or analysed during this study are included in this published article and its supplementary information files.

## Abbreviations

CoA: Coarctation of the aorta
CPB: Cardiopulmonary bypass
MAP: Mean arterial pressure
NIRS: Near-infrared spectroscopy
PaCO_2_: Arterial blood carbon dioxide tension
PDA: Patent ductus arteriosus
SCP: Selective cerebral perfusion
SctO_2_: Cerebral and tissue oxygen saturation
SstO_2_: Somatic and tissue oxygen saturation
SmvO_2_: Mixed venous blood oxygen saturation
